# Gene Expression Changes during the Gummosis Development of Peach Shoots in Response to *Lasiodiplodia theobromae* Infection Using RNA-Seq

**DOI:** 10.3389/fphys.2016.00170

**Published:** 2016-05-09

**Authors:** Lei Gao, Yuting Wang, Zhi Li, He Zhang, Junli Ye, Guohuai Li

**Affiliations:** ^1^Key Laboratory of Horticultural Plant Biology, Ministry of Education, Huazhong Agricultural UniversityWuhan, China; ^2^State Key Laboratory of Crop Stress Biology in Arid Areas, College of Horticulture, Northwest Agriculture and Forestry UniversityYangling, China

**Keywords:** gummosis, *Prunus*, *L. theobromae*, RNA-Seq, defense response, glycometabolism

## Abstract

*Lasiodiplodia theobromae* is a causal agent of peach (*Prunus persica* L.) tree gummosis, a serious disease affecting peach cultivation and production. However, the molecular mechanism underlying the pathogenesis remains unclear. RNA-Seq was performed to investigate gene expression in peach shoots inoculated or mock-inoculated with *L. theobromae*. A total of 20772 genes were detected in eight samples; 4231, 3750, 3453, and 3612 differentially expressed genes were identified at 12, 24, 48, and 60 h after inoculation, respectively. Furthermore, 920 differentially co-expressed genes (515 upregulated and 405 downregulated) were found, respectively. Gene ontology annotation revealed that phenylpropanoid biosynthesis and metabolism, uridine diphosphate-glucosyltransferase activity, and photosynthesis were the most differentially regulated processes during gummosis development. Significant differences were also found in the expression of genes involved in glycometabolism and in ethylene and jasmonic acid biosynthesis and signaling. These data illustrate the dynamic changes in gene expression in the inoculated peach shoots at the transcriptome level. Overall, gene expression in defense response and glycometabolism might result in the gummosis of peach trees induced by *L. theobromae*.

## Introduction

Fungal gummosis of peach (*Prunus persica* L.) trees was first reported in 1974 in Central Georgia (Weaver, 1974). Three species of *Botryosphaeria* fungus cause this disease, namely, *Lasiodiplodia theobromae, Diplodia seriata*, and *Fusicoccum aesculi* were identified and reported in follow-up studies (Britton and Hendrix, 1982; Wang et al., 2011). Previous studies suggested that the pathogenicity of *L. theobromae* JMB-122 was stronger than other species (Wang et al., 2011). The hyphae of *L. theobromae* were observed on the phloem of peach shoots at 2 days post-inoculation (Li et al., 2014b). In cashew gummosis caused by *L. theobromae*, hyphae were often colonized in the rays, vessels and parenchyma cells (Muniz et al., 2011). *Botryosphaeria* spp. are capable of degrading lignin and pectin (Alves da Cunha et al., 2003). Degradation of cell walls was observed in peach shoots and cashew branches infected by *L. theobromae* (Muniz et al., 2011; Li et al., 2014b). In addition, *L. theobromae* induced the expression of cell wall degrading-related genes and triggered cell death of the inoculated peach shoots (Li et al., 2014b). A serious case of peach gummosis can cause tree death, which significantly affects agronomy and economics (Beckman et al., 2003; Wang et al., 2011). The main symptom of peach gummosis is gum exudation from tree trunks, branches, and fruits. The main components of gum are polysaccharides (Simas et al., 2008; Simas-Tosin et al., 2010). A recent study has observed polysaccharide accumulation and investigated carbohydrate metabolism changes in peach shoots infected with *L. theobromae* (Li et al., 2014a). These results suggest that glycometabolism directly relates to peach gum formation.

Pathogen attack in plants alters the levels of various secondary metabolites, among which, phenylpropanoid compounds contribute to pathogen resistance (Dixon et al., 2002). Plant hormones such as ethylene (ET) and jasmonates [mainly jasmonic acid (JA) and methyl jasmonate (JA-ME)] are essential factors in gum formation. Ethephon (ETH, 2-chloroethylphosphonic acid, ET-releasing compound) can induce sour cherry gummosis (Olien and Bukovac, 1982), and ET can initiate gum duct formation in almond fruits (Morrison et al., 1987). The application of ET in tulip bulbs leads to gum formation, and the effect can be prevented through pretreatment with the ET receptor inhibitor 1-methylcyclopropane (1-MCP) (de Wild et al., 2002). JA can induce gummosis in tulip (Skrzypek et al., 2005a,b) and grape hyacinth (Miyamoto et al., 2010), as well as in various species of stone-fruit trees such as plum shoots and fruits (Saniewski et al., 2002), apricot (Saniewski et al., 2001), and peach shoots (Saniewski et al., 1998; Li et al., 2015).

High-throughput sequencing technologies have been recently developed for transcriptome profiling, referred to as RNA-Seq (Wang et al., 2009; Marguerat and Bähler, 2010). RNA-Seq has been widely applied to study plant diseases caused by bacteria (Kim et al., 2011; Socquet-Juglard et al., 2013), fungi (Xu et al., 2011; de Jonge et al., 2012; Kunjeti et al., 2012; Windram et al., 2012; Czemmel et al., 2015), and viruses (Zhang et al., 2012; Rubio et al., 2015) because of its capacity to elucidate the molecular mechanism underlying plant-pathogen interactions.

The molecular mechanism underlying peach fungal gummosis remains unclear to date. Thus, we used high-throughput Illumina sequencing in the present study to analyze the transcriptome of peach shoots at 12, 24, 48, and 60 h after inoculation (HAI) with *L. theobromae*. We analyzed differentially expressed genes (DEGs) and their significantly enriched pathways after pathogen infection, and discussed possible factors influencing gummosis development. The global view of the host transcriptional changes could contribute to our understanding of gum symptom development in peach shoots infected with *L. theobromae*.

## Materials and methods

### Plant material and pathogen material

Peach plants (*P. persica* L. “Spring Snow”) was grafted onto wild peach rootstocks and cultivated in the experiment field of Huazhong Agricultural University (Wuhan, Hubei Province, China). Current-year shoots approximately 6 mm in diameter were collected from 4-year-old peach plants in 2012. *L. theobromae* strain JMB-122 was isolated from Hubei Province, China (Wang et al., 2011). Before inoculation, *L. theobromae* JMB-122 was cultured on potato dextrose agar (PDA) medium at 28°C for 3 days.

### Inoculation of peach shoots with *L. theobromae*

The inoculation method was based on a previous study (Li et al., 2014b). In brief, after surface-sterilized peach shoots were cut into 15 cm-long segments and then wounded with a sterilized needle. A single mycelial plug (4 mm in diameter) of *L. theobromae* was placed onto the wound point. Shoot segments inoculated with sterile PDA medium without *L. theobromae* were treated as controls. The inoculated and control shoots were placed in glass bottles containing 100 mL of sterilized water. The shoots and glass bottles were covered with clear plastic wrap and then placed in a light incubator at 28°C, 90% relative humidity with a photoperiod of 12/12 h light (20,000 lux) /dark.

### Measurement of ET production

The inoculated and mock-inoculated shoots were used to measure the ET production rate. The mycelial plug or the PDA medium was removed before the shoots were placed in a 500 mL Erlenmeyer flask. Each Erlenmeyer flask containing about 15 shoots was sealed airtight by a rubber stopper. The shoots were sealed for up to 6 h at 28°C at 0, 1, 2, 3, and 4 days after inoculation. Then, 1 mL gas was extracted from the airtight Erlenmeyer flask by using gas-tight syringes. ET was detected using a gas chromatograph (Agilent, 7890A, USA) equipped with a DB-624 column and a flame ionization detector (FID). The injection, FID, and column temperature was 250°C, 250 and 40°C, respectively. The pressure in the column was 2.8109 Pa. The carrier gas was pure nitrogen (N_2_) with a rate of 16 mL·min^−1^. The external standard method was used in this study; the retention time of the standard sample (from Newradar special GAS Co., Ltd., China) and the peak area were used as qualitative and quantitative data, respectively. The rate of ET production was expressed as μL·kg^−1^·h^−1^. The results of ET production rate are shown as the means ± SD of three independent biological replicates.

### Plant sample preparation and RNA preparation

Peach shoot tissues were collected within a 0.5–1.0 cm range from the wound point of the inoculated shoots (J) and the mock-inoculated shoots (C) at 12, 24, 48, and 60 HAI. The samples were immediately frozen in liquid nitrogen and stored at −80°C. Both infected and control samples were collected from eight peach shoots in a randomized manner. The J and C samples at 12, 24, 48, and 60 HAI were used to extract RNA. The total RNA was extracted using the EASYspin Plus RNA kit (Aidlab, Beijing, China). Any genomic DNA was removed by DNAase (TaKaRa, Dalian, China). The RNA yield and purity were checked through NANODROP 2000 (Thermo, USA), and RNA integrity was verified through electrophoresis on 1.5% agarose gel.

### cDNA library construction and RNA-Seq

The eight RNA samples were sent for RNA-Seq using the Illumina Genome Analyzer at ABLife (Wuhan, China) in 2012. For each sample, 10 μg of total RNA was used for RNA-Seq library preparation. Polyadenylated mRNAs were purified and concentrated with dT-conjugated magnetic beads (Invitrogen) before used for directional RNA-Seq library preparation. The purified mRNAs were iron-fragmented at 95°C followed by end repair and 5′ adaptor ligation. Then, reverse transcription was performed with RT primer harboring 3′ adaptor sequence and randomized hexamer. The cDNAs were purified and amplified, and PCR products corresponding to 200–500 bp were purified, quantified, and stored at −80°C until used for sequencing. The libraries for high-throughput sequencing were prepared following the manufacturer's protocol and then applied to the Illumina GAIIx system for 80-nucleotide single-end sequencing. Raw data were collected by the sequencer. Reads containing two N were removed, the adaptor was trimmed on the basis of adapter information, and low-quality reads were trimmed. After these steps, reads with lengths ≥20 nt were considered clean.

### Mapping reads to the genome and identification of differentially expressed genes (DEGs)

The *P. persica* v1.0 genome dataset was used as a reference. The abundance of each gene was normalized to reads per kilo bases per million reads (RPKM) for between-sample comparison purposes. The edgeR software was applied to identify DEGs. Fold change (|log_2_FC| ≥ 1) and *p*-value (*p* ≤ 0.01) were used as statistical significance indexes.

### Validation of RNA-Seq analysis by quantitative real-time polymerase chain reaction (qRT-PCR)

First-strand cDNA was synthesized from 1.0 μg of RNA using oligo (dT) primers by using a PrimeScript® RT Reagent Kit with gDNA Eraser (TaKaRa, Dalian, China) in accordance with the manufacturer's protocol. The cDNA was diluted to a final concentration of 300 ng·μL^−1^ and used as the template for qRT-PCR. qRT-PCR was performed on the LightCyler® 480 real-time detection system (Roche Diagnostics, Switzerland). The intercalation dye SYBR Green (TransStart®) was used as a fluorescent reporter. Translation elongation factor 2 was used as a reference gene to normalize gene expression in according with a previously published report (Sherif et al., 2012). In brief, 15 μL of the PCR system contained 300 ng of cDNA, 10 mmol of each primer, and 7.5 μL of 2 × TransStart® Top Green qPCR SuperMix (TransGen, Beijing, China). The reaction was performed at 95°C for 30 s, followed by 45 cycles of 95°C for 5 s, 60°C for 30 s, 72°C for 30 s. Relative gene expression was calculated using the comparative 2^−Δ*ΔCT*^ method (Livak and Schmittgen, 2001). The qRT-PCR results are shown as the means ± SD of three independent biological replicates.

## Results

### Symptom changes in peach shoots infected with *L. theobromae*

**Figure 1:**
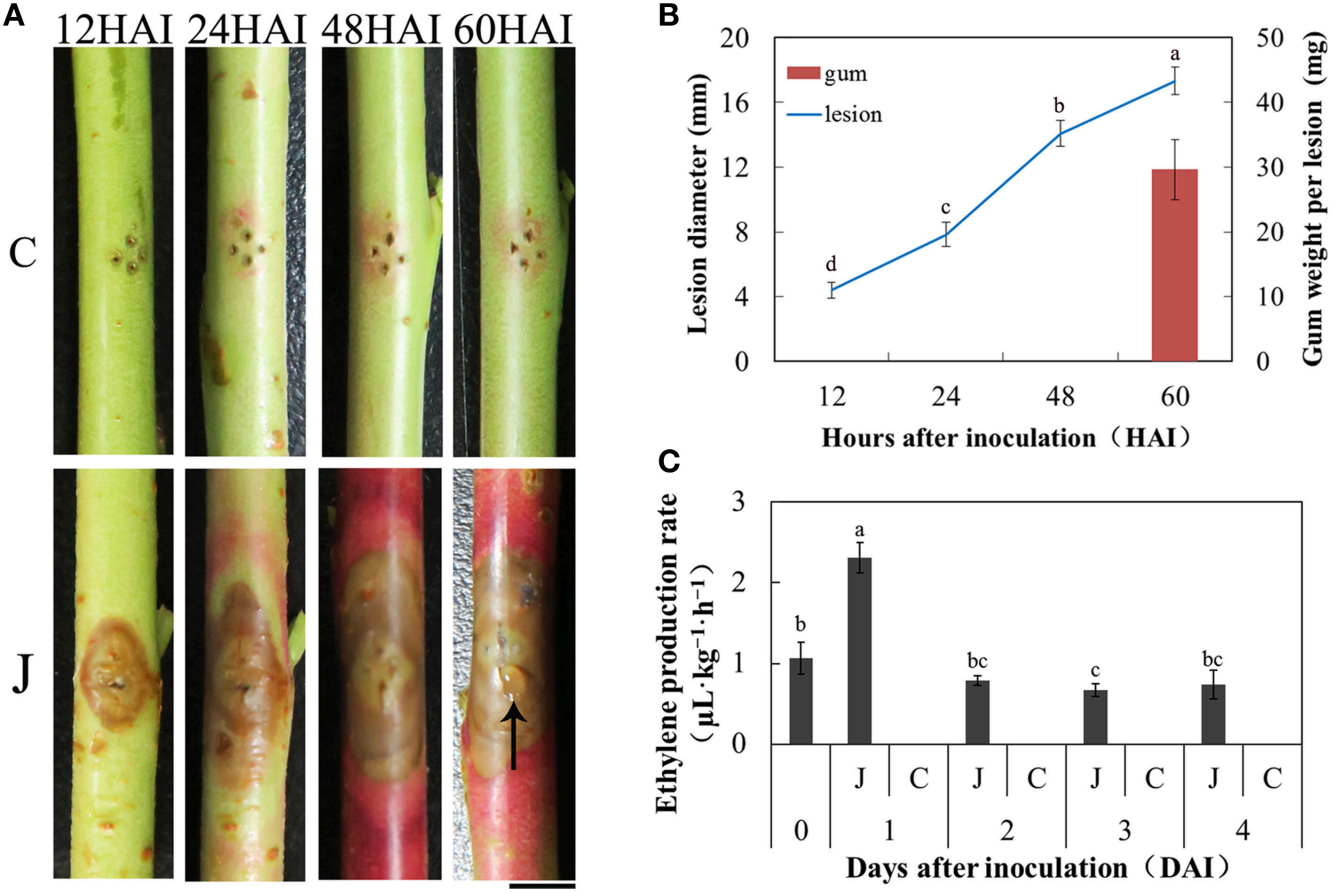
**Peach gummosis development and ethylene production rate. (A)** Development of symptoms of peach current-year detached shoots inoculated with *L. theobromae*. C, mock-inoculated peach shoots; J, inoculated peach shoots; HAI, hours after inoculation; The black arrow indicates gum exudation; Bar is 5 mm. **(B)** Dynamic changes in lesion diameter at different hours and gum weight per lesion at 60 HAI. The values presented are the means ± SD of six independent determinations. **(C)** ET production rate of the inoculated and mock-inoculated current-year peach shoots. At 0 days, the shoots were only wounded. In the days following, no ET was detectable in control shoots. Data were analyzed by ANOVA using SAS program package (version 8.1; SAS Institute, Cary, NC) to determine differences in lesion diameter **(B)** and ET production rate **(C)**. Means with the same letters are not significantly different at the 5% level by Duncan's multiple range test.

A typical symptom observed in the inoculated peach shoots was the increased lesions compared with the mock-inoculated peach shoots. At 12 HAI, the lesion diameter was about 5 mm. From 24 HAI to 48 HAI, lesions developed at a comparatively rapid speed. The lesions were 17.3 ± 1.0 mm at 60 HAI (Figure 1B). Another typical symptom was the distinct red color surrounding the lesions at the beginning of 24 h. Importantly, the gum exudation was visible on the inoculated point at 60 h (Figure 1A), with a 29.3 ± 4.7 mg gum weight (Figure 1B) per inoculated shoot in average. When peach shoots were inoculated with *L. theobromae*, the maximum production rate of ET was reached at 1 day, but this rate declined during 2–4 days. ET production in the control plants on days 1–4 fell lower than the minimum detectable concentration. Thus, gas chromatography failed to generate any efficient data.

### Analyses of RNA-Seq data

In total, eight cDNA preparations were sequenced. The number of raw reads produced for each library exceeded 10 million (Table 1). After filtering, most of the clean reads were still more than 80% of the raw data, except C12 (66.15%) and C24 (79.24%). The useful length of the vast majority of the sequence was 66–67 bp, indicating few number of low-quality bases. The trimmed RNA-Seq reads were mapped on the v1.0 *P. persica* reference genome. Approximately 72–81% of the clean reads were mapped to the genome, except C12 (59.20%). The unique mapped reads accounted for more than 94% of the total mapped reads, indicating the low proportion of rRNA contamination. The clean reads were distributed mainly (an average of 75.1%) in the coding sequence of the genomic regions (Supplementary Table 1). A total of 20771 genes were detected as being expressed using the eight samples, or 74.58% of the total 27852 predicted genes of the v1.0 peach genome (Verde et al., 2013). In general, the RPKM value of the 60–70% reads of each sample was below 20, indicating a greater proportion of lowly expressed genes than highly expressed genes.

**Table 1:** **Summary of RNA-Seq data collected from the inoculated (J) and the mock-inoculated (C) peach shoots at the four selected hours after inoculation (HAI) and assemblies**.

**Sample**		**Raw reads**	**Clean reads**	**Reads mapped *P. persica* v 1.0**	**Unique mapped**
12 HAI	J	10620646	8763947 (82.52%)	6320708 (72.12%)	6012821 (95.13%)
	C	14403060	9527620 (66.15%)	5640474 (59.20%)	5360326 (95.03%)
24 HAI	J	12457518	10567109 (84.83%)	7859332 (74.38%)	7501716 (95.45%)
	C	10672969	8456836 (79.24%)	6083248 (71.93%)	5774232 (94.92%)
48 HAI	J	13085397	11608410 (88.71%)	9428886 (81.22%)	8998979 (95.44%)
	C	12063922	9 888030 (81.96%)	7513771 (75.99%)	7140544 (95.03%)
60 HAI	J	12290504	10417691 (84.76%)	8248739 (79.18%)	7869553 (95.40%)
	C	10015740	8434545 (84.21%)	6604147 (78.30%)	6306888 (95.50%)

### DEGs in J and C

**Figure 2:**
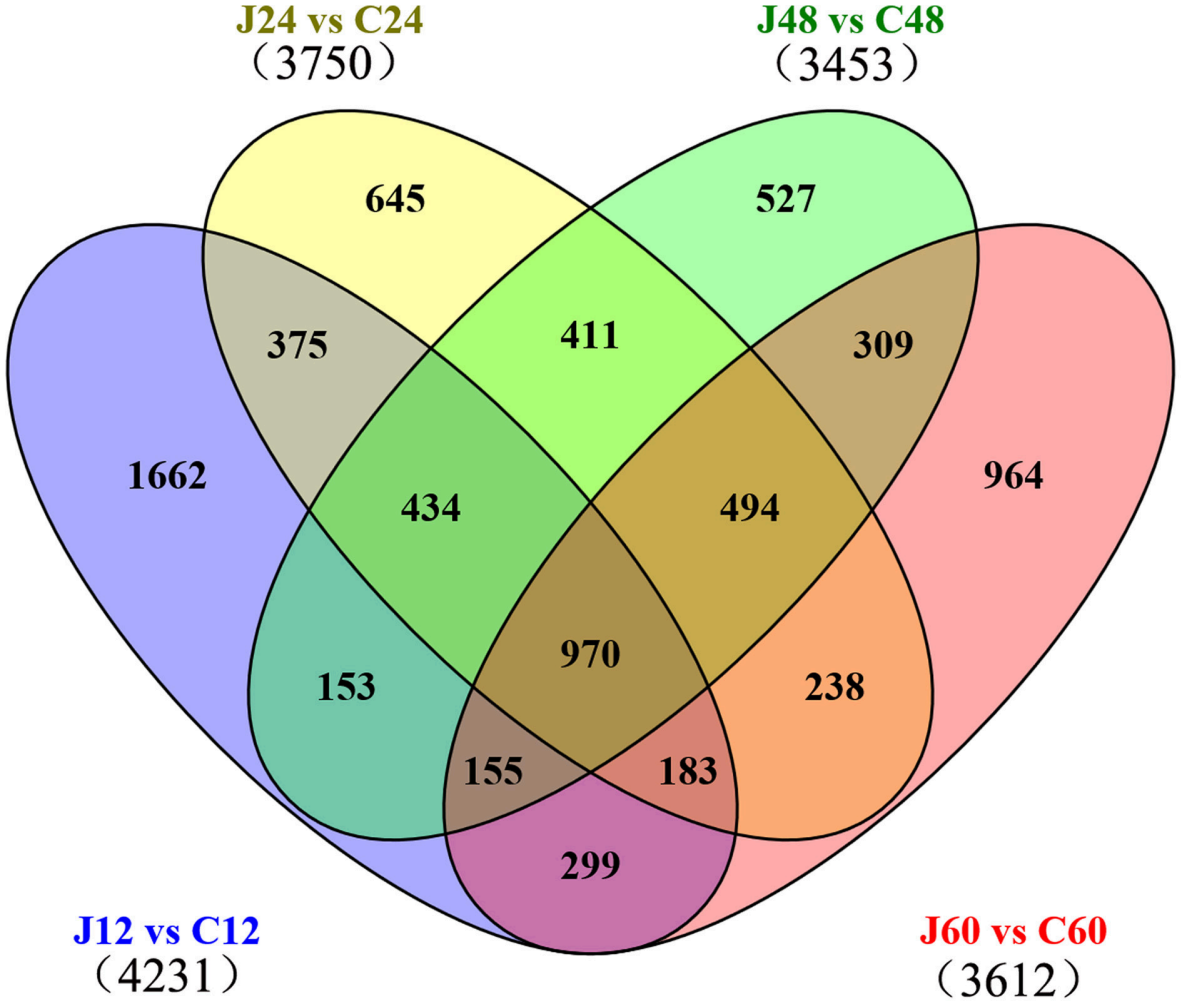
**Venn diagram of differentially expressed genes among the four comparisons performed (J12 vs. C12, J24 vs. C24, J48 vs. C48, and J60 vs. C60)**. “J12 vs. C12” means a comparison between the inoculated (J) and mock-inoculated (C) peach shoots at 12 h after inoculation.

The selection standards for DEGs are the fold change ≥2 or ≤ −2 and *p* ≤ 0.01 (Supplementary Figure 1). Of the 20771 genes detected in this transcriptome, 4231, 3750, 3453, and 3612 were differentially expressed at *p* ≤ 0.01 and |log_2_FC| ≥ 1 between J12 and C12, J24 and C24, J48 and C48, and J60 and C60, respectively. Then, 515 and 405 DEGs were co-upregulated and co-downregulated among the four comparisons, respectively (Table 2; Supplementary Table 2). The number of upregulated genes was less than that of downregulated genes between J24 and C24, and J60 and C60. The top 10 upregulated and top 10 downregulated genes in each of the four post-inoculated stages are listed in the Supplementary Table 3. A total of 1662, 645, 527, and 964 DEGs were specific for J12 vs. C12, J24 vs. C24, J48 vs. C48, and J60 vs. C60, respectively (Figure 2; Supplementary Table 4). In addition, the number of upregulated genes was almost the same as that of the downregulated genes for J12 vs. C12, J24 vs. C24, and J48 vs. C48, respectively. However, in the comparison of J60 vs. C60, 288 (30%), and 676 (70%) of the 964 specific DEGs were up- and downregulated, respectively (Table 2).

**Table 2:** **Distribution of differentially expressed genes (DEGs) between the inoculated (J) and control (C) peach shoots at each of the four selected hours after inoculation (HAI)**.

	**J12 vs. C12**	**J24 vs. C24**	**J48 vs. C48**	**J60 vs. C60**	**J vs. C**
Total DEGs	4231	3750	3453	3612	920
Upregulated	2257	1860	1768	1437	515
Downregulated	1974	1980	1685	2175	405
Shared DEGs	2569	3105	2926	2648	
Specific DEGs	1662	645	527	964	
Upregulated	880	360	248	288	
Downregulated	782	285	279	676	

*J12 vs. C12 indicates a comparison between J and C at 12 HAI; J vs. C represents the co-DEGs (co-upregulated and co-downregulated) among the four comparisons performed*.

### Functional analysis of DEGs in J and C

The Database for Annotation, Visualization and Integrated Discovery (DAVID) online platform (https://david.ncifcrf.gov/) was used to analyze the function of the DEGs. The up- and down-regulated cluster groups of the DEGs were subjected to gene ontology (GO) term analysis. Among the upregulated clusters, the DEGs were significantly enriched in “phenylpropanoid biosynthetic and metabolic process.” Glycosyltransferase, especially the relevant uridine diphosphate (UDP)-glucosyltransferase genes were highly enriched from 24 h in the inoculated shoots. At 12 HAI, the genes involved in JA biosynthesis were highly enriched. Among the downregulated clusters of the GO term analysis, chloroplast, plastid, and photosynthesis were significantly enriched (Supplementary Table 5). In the comparison of “J12 vs. C12,” the metabolic process of starch, glucan, and polysaccharides was downregulated. Furthermore, the expression levels of relevant DEGs controlling the catabolic process of starch, glucan, and polysaccharide were downregulated in the inoculated shoots (detailed data in Supplementary Table 6). The same tendency was also observed on three other comparisons.

**Figure 3:**
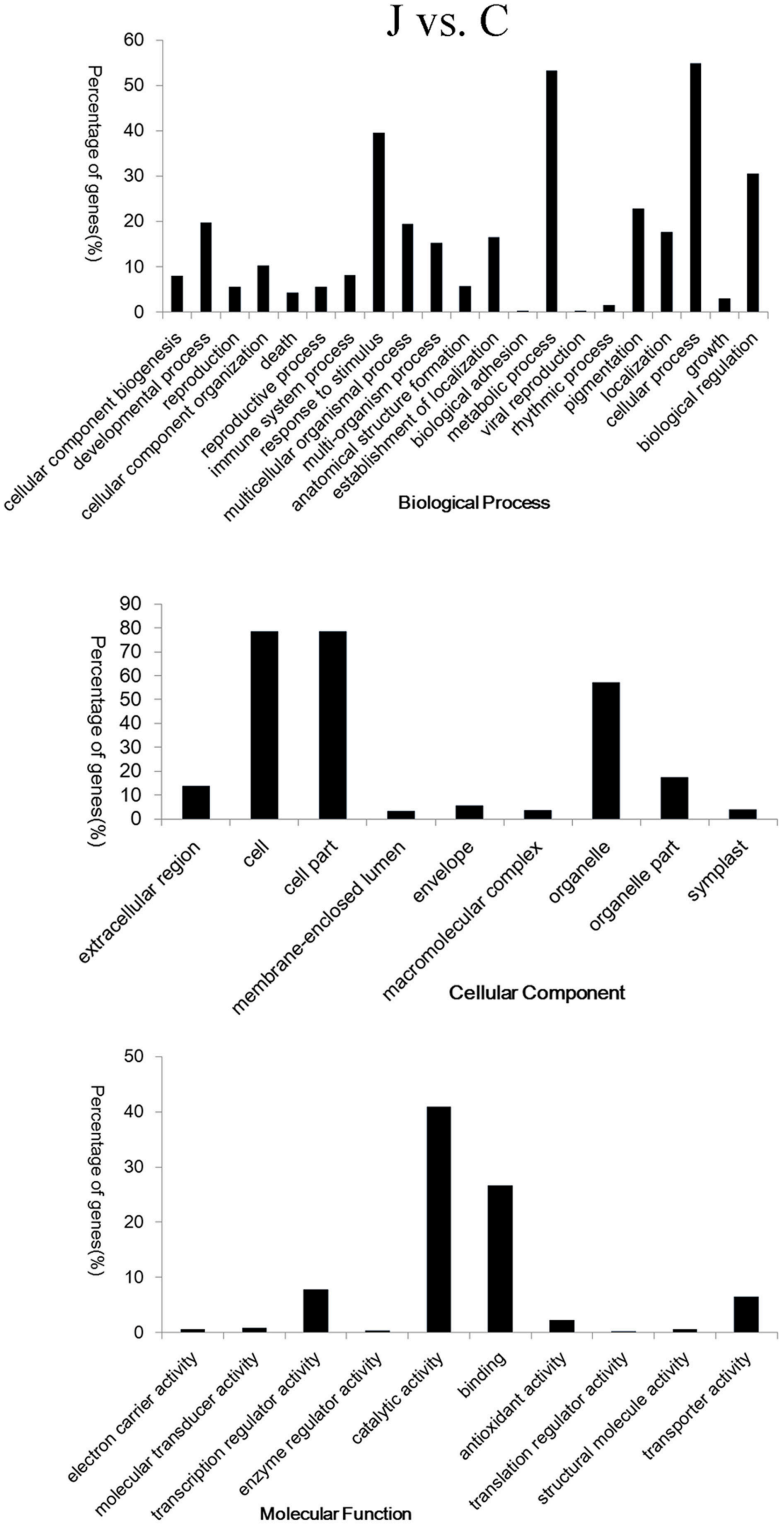
**Gene ontology (GO) classification of the 920 co-differentially expressed genes in the inoculated (J) and mock-inoculated (C) peach shoots**. Annotations are grouped by biological process, cellular component, and molecular function. The percentage of genes (%) is listed for each category.

GO terms were assigned to gain an overall understanding of the 920 DEGs identified in the J vs. C analysis. The broad categories for the three major GO functional domains (biological process, cellular component, and molecular function) are shown in Figure 3. The categories “metabolic process,” “cellular process,” “response to stimulus,” “biological regulation,” and “pigmentation” were the five representative categories based on the biological process (406 DEGs). The categories “cell,” “cell part,” “organelle,” “organelle part,” and “extracellular region,” captured most of these genes based on cellular component (416 DEGs), and the categories “catalytic activity,” “binding,” “transcription regulator activity,” and “transporter activity” captured most of these genes based on molecular function (442 DEGs) (Figure 3). Detailed information was obtained through the DAVID online platform (Supplementary Table 5). The terms “phenylpropanoid biosynthetic and metabolic process,” “oxidation reduction,” “UDP-glucosyltransferase activity,” and “carbohydrate transport and metabolism or signal transduction mechanisms” were significantly enriched in the upregulated clusters. Photosynthesis was obviously inhibited in the inoculated peach shoots.

#### *L. theobromae* infection significantly increased the expression of genes involved in biosynthesis and metabolism of phenylpropanoid and the activity of UDP-glucosyltransferase

**Figure 4:**
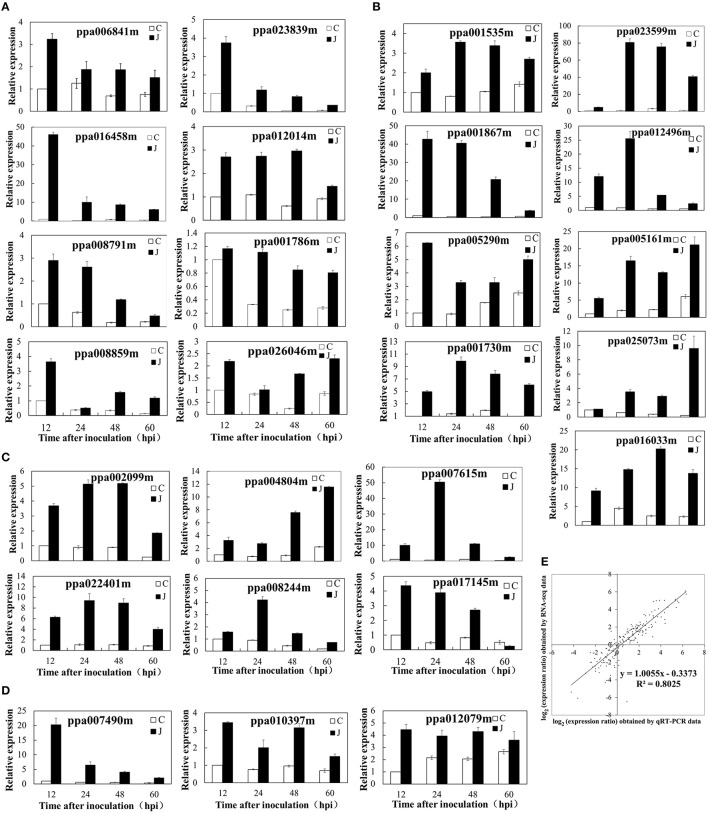
**Validation of differential expression by quantitative real-time polymerase chain reaction (qRT-PCR)**. **(A)** Eight genes associated with the ET biosynthetic/metabolic process or signaling pathway. **(B)** Nine genes related to UDP-glucuronosyl/UDP-glucosyltransferase activity, carbohydrate transport, and metabolism or signal transduction mechanisms. **(C)** Six genes related to the biosynthetic or metabolic process of phenylpropanoids. **(D)** Three genes responded to JA biosynthesis. **(E)** Comparison between the gene expression ratios obtained from RNA-Seq data (y-axis; log_2_) and qRT-PCR (x-axis; log_2_).

The qRT-PCR analysis of several genes (Figure 4C) and the heat map diagram of DEGs involved in phenylpropanoid biosynthesis and metabolism (Supplementary Figure 2) also revealed the same result. Higher patterns of expression were exhibited by genes involved in the anthocyanin biosynthetic pathway. These genes include phenylalanine ammonia lyase (ppa002099m), cinnamate-4-hydroxylase (ppa018282m and ppa004544m), 4-coumarate: CoA ligase (ppa003854m and ppa022401m), chalcone synthase (ppa006888m, ppa006899m, ppa008402m, and ppa023080m), chalcone-flavanone isomerase (ppa011276m), flavanone 3-hydroxylase (ppa007636m), dihydroflavonol 4-reductase (ppa008069m), and leucoanthocyanidin dioxygenase (ppa007738m) (Figure 4C; Supplementary Table 8). An overview of genes involved in anthocyanin biosynthetic pathway was presented in Supplementary Figure 3. Genes encoding galactosyltransferase (ppa006755m, ppa022137m, and ppa015950m), UDP-glycosyltransferase and UDP-glucosyltransferase were upregulated several to hundreds-fold in the inoculated peach shoots compared with the control shoots (Supplementary Table 9).

#### Genes related to carbohydrate metabolism are differentially expressed during *L. theobromae* infection

Cellulose and pectin are the main components of plant cell walls. The expression levels of ppa004653 and ppa004719m (*cellulase* genes) increased in the inoculated peach shoots compared with those in the control shoots at 24 HAI (2- and 4.6-fold, respectively) and 48 HAI (7.5- and 2.8-fold, respectively). Ppa000557m (*cellulose synthase 6*) was downregulated at 12 HAI. However, several cellulose synthase-like genes were upregulated simultaneously (Supplementary Table 10). The expression of most of the pectin lyase-related genes was promoted within 12–48 HAI in the inoculated peach shoots. Interestingly, pectin methylesterase inhibitor-related genes were almost upregulated as well. At 60 HAI, the number of downregulated genes increased. Meanwhile, ppa007271m (gene encoding pectin lyase-like superfamily protein) was significantly upregulated at all HAI periods (Supplementary Table 10).

A comprehensive illustration of carbohydrate metabolism based on the transcriptomic changes during *L. theobromae* infection is presented in Supplementary Figure 4. The expression levels of Sucrose synthase (SS) genes (ppa001535m, ppa017606m, and ppa001135m) were higher in the inoculated peach shoots than in the control shoots. At 60 HAI, ppa001535m, and ppa017606m were downregulated in the inoculated peach shoots. Sucrose phosphate synthase (SPS) genes were also upregulated (ppa000622m) or showed this tendency (ppa000636m and ppa000639m) at 60 HAI. The expression levels of *glucose 6-phosphate translocator* genes (ppa006608m and ppa006795m) increased in the inoculated peach shoots (Supplementary Figure 5). Ppa007136m (*alpha-galactosidase 2*) was downregulated in the inoculated peach shoots, indicating that galactose hydrolysis was inhibited. Meanwhile, the expression of ppa008032 (*UDP-D-glucose 4-epimerase 5*) was promoted. UDP-D-glucose 4-epimerase (UGE) is the key enzyme in UDP-D-galactose biosynthesis (Seifert et al., 2002; Rösti et al., 2007). The expression levels of ppa008317m (*UDP-xylose synthase 4*) and ppa001692m (*beta-D-xylosidase 4*) were also enhanced, especially at 48 HAI. Higher patterns of expression were observed in genes involved in the catabolism of fructose in the inoculated peach shoots relative to the control shoots. These genes include *hexokinase* (ppa004471m), *phosphofructokinase* (ppa003994m and ppa004086m), and *mannose-6-phosphate isomerase* (ppa005846m). However, ppa007744m, ppa025195m, and ppa006746m (encoding aldolase) were downregulated. This result indicates that the further decomposition of fructose-1, 6-bisphosphate was inhibited in the inoculated peach shoots. Fructose-6-bisphosphate can participate in the biosynthesis of D-mannose-6-phosphate (Supplementary Figure 6). In addition, genes related to the degradation of glycosaminoglycan and other glycans were downregulated in the inoculated peach shoots.

#### Genes involved in ET and JA biosynthesis and signaling were mainly upregulated in the inoculated peach shoots

An overview of genes involved in ET and JA biosynthetic pathway was presented in Supplementary Figure 7. The *SAM synthetase* (ppa006841m) was overexpressed in the inoculated peach shoots. Higher patterns of expression were also observed (RPKM values) or obtained (qRT-PCR values) in pivotal genes related to the ET biosynthesis. These genes include *1-aminocyclopropane-1-carboxylate* (*ACC*) *synthase* 6 (ppa016458) and ET-forming enzyme (ppa008791m) (Supplementary Figure 8A; Figure 4A). The expression pattern of ppa016458 and ppa008791m was consistent with the variation trend of ET production. Several DEGs encoding ET signaling components have also been identified. The expression levels of ppa023839m and ppa012014m (genes encoding ET response factor) and ppa001786m (ET sensor) determined by qRT-PCR were increased several times to 10 times after *L. theobromae* infection, this result is consistent with the RNA-Seq data (Figure 4A). Other DEGs involved in the ethylene signaling pathway were listed in Supplementary Figure 8A. The protein involved in JA biosynthesis (12-oxophytodienoate reductase 2, ppa007490m) was overexpressed in the inoculated peach shoots. The expression levels (RPKM values) of other genes, such as *allene oxide synthase* (ppa025045m), *allene oxide cyclase 3* (ppa010397m), and *allene oxide cyclase 4* (ppa012079m) were higher (two to five-fold) in the inoculated peach shoots than in the control shoots at 12, 24, and 48 HAI, although no difference (ppa010397m and ppa012079m) or lower expression (ppa025045m) was observed at 60 HAI. JA-ME is catalyzed by JA carboxyl methyltransferase, this gene (ppa017829m) was upregulated at 12 and 24 HAI and downregulated at 48 and 60 HAI in the inoculated peach shoots (Supplementary Figure 8B). The qRT-PCR results were similar to the RPKM values (Figure 4D).

### Verification of gene expression profiles using qRT-PCR

At 12, 24, 48, and 60 HAI, we collected samples from eight shoots and pooled them for RNA extraction and subsequent RNA-Seq analysis. A one-to-one correspondence exists between J and C in this experiment. To confirm the accuracy and reproducibility of the transcriptome analysis results, 26 representative genes were selected for real-time qRT-PCR validation in a separate experiment. The primers of these genes are shown in Supplementary Table 7, and the The qRT-PCR results are shown in Figure 4. The fold change of the gene expression ratios between RNA-Seq and qRT-PCR was analyzed by linear regression. The overall correlation coefficient was 0.8025, indicating the reliability of the RNA-Seq data (Figure 4E).

## Discussion

### Phenylpropanoid metabolism and glycosyltransferase activity of the inoculated peach shoots

In general, a marked induction of genes is involved in the biosynthesis of phenylpropanoids in plants as a response to pathogens (Shetty et al., 2011; Xu et al., 2011; Kostyn et al., 2012; Muñoz-Bodnar et al., 2014). Phenylpropanoids play important roles in plant resistance to pathogen attacks (Dixon et al., 2002; Korkina, 2007; Naoumkina et al., 2010; Boubakri et al., 2013). In addition, the protective action of phenylpropanoids in plants is assumed to be based on their antioxidant and free radical scavenging properties. Flavonoids are representative substances of phenylpropanoid derivatives in plants (Tahara, 2007). Flavonoids are natural defense compounds in plants against pathogens. Anthocyanins represent one class of flavonoids that are significantly accumulated around the lesions of the inoculated peach shoots, resulting in a red coloration on infected peach shoots (Figure 1A; Li et al., 2014b). As important secondary metabolites, anthocyanins contribute to protect plants against pathogenic attack (Winkel-Shirley, 2001). As a type of abiotic stress, wounds can also induce the flavonoids accumulation. Therefore, a slight red coloration appeared around the wound site of the control shoots (Figure 1A). However, the effect was much less than the stress response induced by *L. theobromae*. Plant UDP-glycosyltransferases and UDP-glucosyltransferases can be involved in the modification of phenylpropanoids (Vogt and Jones, 2000). The glycosylated form of these compounds exhibits enhanced solubility, stability, and transport properties (Li et al., 2001), and can be stored as preformed defense compounds. The glycosylated defense compounds (e.g., flavonoids) involved during pathogen attacks are activated by deglycosylation (Jasiński et al., 2009). Several genes encoding UGTs play important roles in plant defense against pathogens (Chong et al., 2002; Poppenberger et al., 2003; von Saint Paul et al., 2011). For example, UGT73B3 and UGT73B5 supposedly participate in the regulation of redox status and general detoxification of reactive oxygen species and contribute to the resistance of Arabidopsis *to Pseudomonas syringae* pv. *tomato* (Simon et al., 2014).

### Carbohydrate metabolism and gum formation of the inoculated peach shoots

Some pathogens can secrete polygalacturonases and endo-polygalacturonases which enable them to penetrate the host plant by degrading the plant cell wall pectin. *Botryosphaeria* spp. belonging to ascomycetous fungi can degrade lignin and pectin (Alves da Cunha et al., 2003). The pectinase produced by these fungi could degrade the cell wall structures (Srivastava et al., 2013). PMEIs (pectin methylesterase inhibitors) can inhibit pectin methylesterases. Lionetti et al. (2007) reported that PMEIs contributed to the defense of Arabidopsis to *B. cinerea*. In the present study, the transcript levels of genes encoding PMEIs increased, especially during the early and middle infection periods. The defense response of the peach shoot tissues was stimulated by *L. theobromae* infection. However, some genes encoding pectin lyase-like superfamily protein were also upregulated (Supplementary Table 10). Hence, PMEIs could not completely prevent the breakdown of pectin. Overall, the new biosynthesis of cellulose can be assumed to be accompanied by degradation. The monosaccharide components of peach gum polysaccharides are galactose, arabinose, xylose, mannose, and glucuronic acid (Simas et al., 2008; Simas-Tosin et al., 2009). β-1,4-linked glucose, xyloglucan, rhamnogalacturonan I, homogalacturonan, rhamnogalacturonan II, and arabinan are representative components of plant cell wall polysaccharides (Vorwerk et al., 2004). A previous study speculated that peach gum arises from the degradation of parenchyma cells around the periderm and vascular cambium (Biggs and Britton, 1988). Recent research has shown that the cell walls were severely degraded in the lesion of inoculated peach shoots (Li et al., 2014b). A follow-up study indicated that not only the infection site but also the glycometabolism of tissues around the lesion of the inoculated peach shoots greatly contributes to peach gum formation (Li et al., 2014a). Therefore, the degradation of plant cell walls after inoculation with *L. theobromae* is just one reason for gum formation.

The expression changes of starch metabolism-related genes indicate that amylose decomposition was promoted in the inoculated peach shoots while starch synthesis was inhibited. A previous report indicated that the amyloplast was disappeared during later infection (Li et al., 2014b). SS and SPS are key enzymes in sucrose metabolism (Winter and Huber, 2000; Ruan, 2014). SS has a dual function although it was previously believed to play a major role in sucrose cleavage (Chourey and Nelson, 1979; Geigenberger and Stitt, 1993; Heim et al., 1993). The measurement results of sucrose content (Li et al., 2014a) indicated that sucrose decomposition was promoted before 60 HAI and then biosynthesis reaction was increased. At 60 HAI, the peach gum spilled out, and then the increased biosynthesis of sucrose may compensate for the consumed sucrose. Glucose 6-phosphate, provided by the catabolism of sucrose and starch, was an important precursor in the biosynthesis of monosaccharide components of peach gum. The upregulated expression of glucose 6-phosphate translocator will contribute to the biosynthesis of monosaccharide components of peach gum. Our RNA-Seq results revealed that the biosynthesis of UDP-D-galactose, UDP-D-xylose, and D-mannose-6-phosphate was increased in the inoculated peach shoots. Previous study showed that the biosynthesis of UDP-D-arabinose and L-arabinose was also enhanced (Li et al., 2014a).

Glycosyltransferases can catalyze the transfer of sugar residue from an activated nucleotide sugar donor to specific acceptor molecules. This process leads to the formation of glycosidic bonds that play important roles in the biosyntheses of disaccharides, oligosaccharides, polysaccharides, and glycoconjugates (Campbell et al., 1997; Breton et al., 2006). Common glycosyl donors in plants are UDP-glucose (Jones and Vogt, 2001; Jones et al., 2003), UDP-galactose (Ishikura and Mato, 1993; Miller et al., 1999), UDP-rhamnose (Bar-Peled et al., 1991; Jones et al., 2003), UDP-xylose (Martin et al., 1999), and UDP-glucuronate (Sawada et al., 2005). Interestingly, galactose, arabinose, xylose, mannose, and glucuronic acid are the main monosaccharide components of peach gum polysaccharides (Simas et al., 2008; Simas-Tosin et al., 2009). In the present study, the amount of these glycosyl donors (UDP-glucose, UDP-galactose, UDP-xylose, and UDP-arabinose) was increased. The high expression of genes encoding relevant galactosyltransferases in the inoculated peach shoots may promote the biosynthesis of peach gum polysaccharides.

### Involvement of ET and JA in peach gummosis development

ET is an important plant hormone signal in plant–pathogen interactions (Bleecker and Kende, 2000). ET production of plant tissues can be enhanced by pathogen invasion (Penninckx et al., 1998; Cohn and Martin, 2005). Consistent results were also determined in our study (Figure 1C). That is, ET biosynthesis was promoted, especially at 24 HAI. S-adenosylmethionine (SAM) is a general donor of methyl groups in the transmethylation reactions and is also an important precursor substance of ET synthesis. Tsuchisaka and Theologis (2004) reported that wounding the hypocotyl tissue of Arabidopsis induces the expression of *AtACS2, 4, 6, 7, 8*, and *11*. In the present study, in addition to wound-treatments, the pathogen challenge aside from wound treatments promoted the expression of ppa016458, as proven in the endogenous production of ET (Figure 1C). Li et al. (2014c) reported that ETH application on peach shoots pre-inoculated with *L. theobromae* promotes gum formation. ETH treatments accelerated the senescence of peach shoots and rapidly increased the contents of sucrose, glucose, and fructose (Li et al., 2014c), which may promote disease development and facilitate gum formation.

In general, the expression data showed that the genes involved in JA biosynthesis were rapidly induced. A similar result has been observed in Arabidopsis defense against *B. cinerea* (Birkenbihl et al., 2012; Windram et al., 2012). In *Plum pox virus* inoculated peach leaves without visible symptoms, JA biosynthesis and signaling genes were upregulated, indicating that JA stimulate plant defense response (Rubio et al., 2015). Interestingly, JA was first isolated from cultures of the fungus *L. theobromae* (Aldridge et al., 1971). JA biosynthesis in *L. theobromae* is similar to that in plants (Tsukada et al., 2010). Thus, JA might play a role in the interaction between peach shoots and *L. theobromae*. Skrzypek et al. (2005b) pointed out that JA-ME substantially reduces the amount of sucrose and reducing sugars in tulips, contributing to gum formation. Li et al. (2015) also speculated that JA-ME treatments cause new synthesis of polysaccharides.

**Figure 5:**
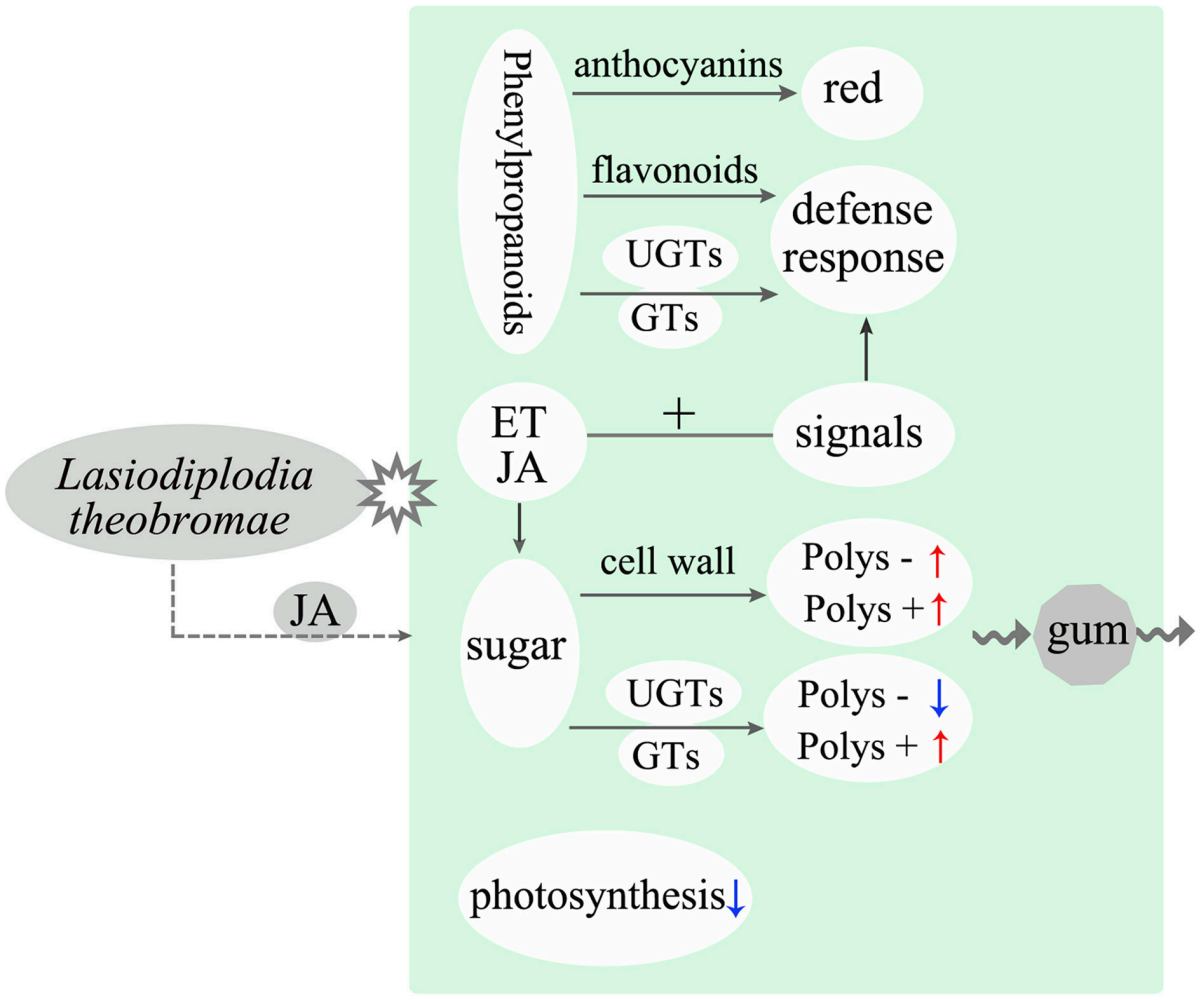
**Model illustrating the main molecular response and gum polysaccharide formation of peach shoots infected with ***L. theobromae*****. “Polys –” means polysaccharide degradation. “Polys +” indicates polysaccharide biosynthesis. Red and blue arrows represent upregulation and downregulation, respectively. Dotted arrow shows a supposed interaction.

In conclusion, inoculation with *L. theobromae* can induce typical gummosis on current-year peach shoots *in vitro*. We analyzed gene expression changes of the peach shoots at different phases after inoculation through RNA-Seq. The main results of this study are summarized in Figure 5. Plant tissues usually exhibit strong defense responses during *L. theobromae* infection. Genes related to the glycometabolism of the inoculated peach shoots were activated, indicating that polysaccharide biosynthesis was increased. In addition, the expression of genes involved in the degradation of cell walls was promoted, but the degradation of glycosaminoglycan and glycan was inhibited. The above factors might be the main cause of the formation of gum polysaccharides induced by *L. theobromae*. Our study provided insights into the mechanisms of peach gummosis caused by *L. theobromae*. However, the role of gum in peach tree response to pathogen attacks requires further investigation.

## Author contributions

LG and YW were responsible for generating the RNA-seq data and for the interpretation of the data. LG carried out qRT-PCR experiments and measured ethylene content, and drafted the manuscript. GL conceived the study and supervised the research. ZL and JY participated in its design and helped to draft the manuscript. HZ participated in the statistical analyses. All authors approved the final manuscript and approved it for publication.

## Funding

This study was supported by the National Natural Science Foundation of China (Grant No. 31471840) and China Agriculture Research System (Grant No. CARS-31-2-4).

### Conflict of interest statement

The authors declare that the research was conducted in the absence of any commercial or financial relationships that could be construed as a potential conflict of interest.
