# Supplementary material for: Gene Expression Changes during the Gummosis Development of Peach Shoots in Response to Lasiodiplodia theobromae Infection Using RNA-Seq
Source: Front Physiol. 2016 May 9;7:170. doi: 10.3389/fphys.2016.00170 (PMC4861008; doi:10.3389/fphys.2016.00170)
Supplement: Supplementary file 1 [file Table1.DOCX]

**Supplementary Table 1** Distribution of RNA-Seq reads across genomic regions.

| Sample | CDS | 5′ UTR | Intergenic | Intron | 3′ UTR |
| --- | --- | --- | --- | --- | --- |
| C12 | 4257985.51 (79.4%) | 162994.28 (3.0%) | 493843.27 (9.2%) | 144007.07 (2.7%) | 301495.86 (5.6%) |
| C24 | 4281545.07  (74.1%) | 168957.30  (2.9%) | 652179.05  (11.3%) | 174228.27 (3.0%) | 497322.31 (8.6%) |
| C48 | 5142249.28 (72.0%) | 198574.46 (2.8%) | 802661.26 (11.2%) | 202276.69 (2.8%) | 794782.29 (11.1%) |
| C60 | 4732265.76 (75.0%) | 182385.33 (2.9%) | 647427.84 (10.3%) | 172444.35 (2.7%) | 572364.71 (9.1%) |
| J12 | 4477781.15 (74.5%) | 172167.38 (2.9%) | 612006.39 (10.2%) | 159638.08 (2.7%) | 591227.99 (9.8%) |
| J24 | 5653776.97 (75.4%) | 234206.08 (3.1%) | 787052.80 (10.5%) | 207451.42 (2.8%) | 619228.72 (8.03%) |
| J48 | 6738471.03 (74.9%) | 246657.37 (2.7%) | 970345.20 (10.8%) | 235048.60 (2.6%) | 808456.78 (9.0%) |
| J60 | 5916541.69 (75.2%) | 233791.37 (3.0%) | 837404.81 (10.6%) | 193291.62 (2.5%) | 688523.49 (8.7%) |

CDS, coding sequence; 5′ UTR, 5′ untranslated region.

The clean reads were distributed mainly in the coding sequence of the genomic regions
