# Supplementary material for: Gene Expression Changes during the Gummosis Development of Peach Shoots in Response to Lasiodiplodia theobromae Infection Using RNA-Seq
Source: Front Physiol. 2016 May 9;7:170. doi: 10.3389/fphys.2016.00170 (PMC4861008; doi:10.3389/fphys.2016.00170)
Supplement: Supplementary file 11 [file Image1.PDF]

Supplementary Figure

Gene expression changes during the gummosis development of peach shoots in response to *Lasiodiplodia theobromae* infection using RNA-Seq

Lei Gao[1], Yuting Wang[2], Zhi Li[3], He Zhang[4], Junli Ye[5] and Guohuai Li[6]*
*Corresponding author: Guohuai Li; E-mail address: liguohuai@mail.hzau.edu.cn

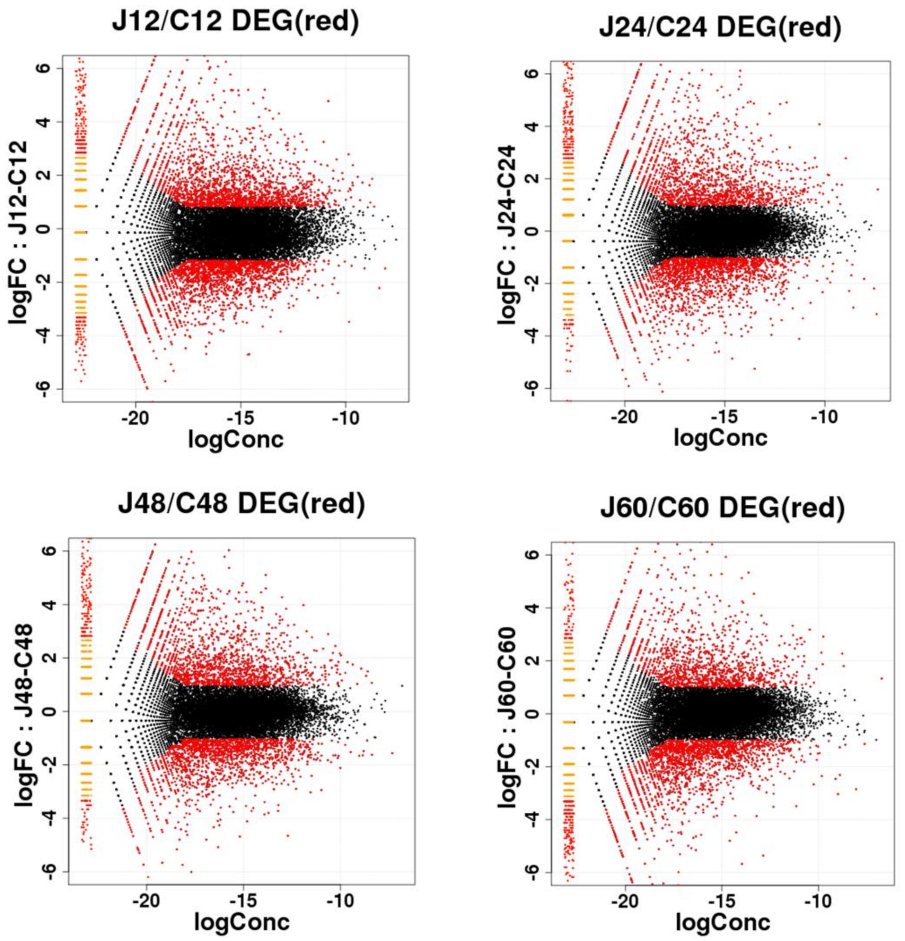

Supplementary Figure 1 Identification of the differentially expressed genes between samples by edgeR program. The selection standards for DEGs are the fold change ≥ 2 or ≤ −2 and $p \leq 0.01$. logConc gives the overall concentration for a gene across the two groups being compared; The smear of points on the left side signifies that genes were observed in only one group of replicate samples.
