# Supplementary material for: Gene Expression Changes during the Gummosis Development of Peach Shoots in Response to Lasiodiplodia theobromae Infection Using RNA-Seq
Source: Front Physiol. 2016 May 9;7:170. doi: 10.3389/fphys.2016.00170 (PMC4861008; doi:10.3389/fphys.2016.00170)
Supplement: Supplementary file 12 [file Image2.PDF]

Supplementary Figure

Gene expression changes during the gummosis development of peach shoots in response to *Lasiodiplodia theobromae* infection using RNA-Seq

Lei Gao[1], Yuting Wang[2], Zhi Li[3], He Zhang[4], Junli Ye[5] and Guohuai Li[6]*
*Corresponding author: Guohuai Li; E-mail address: liguohuai@mail.hzau.edu.cn

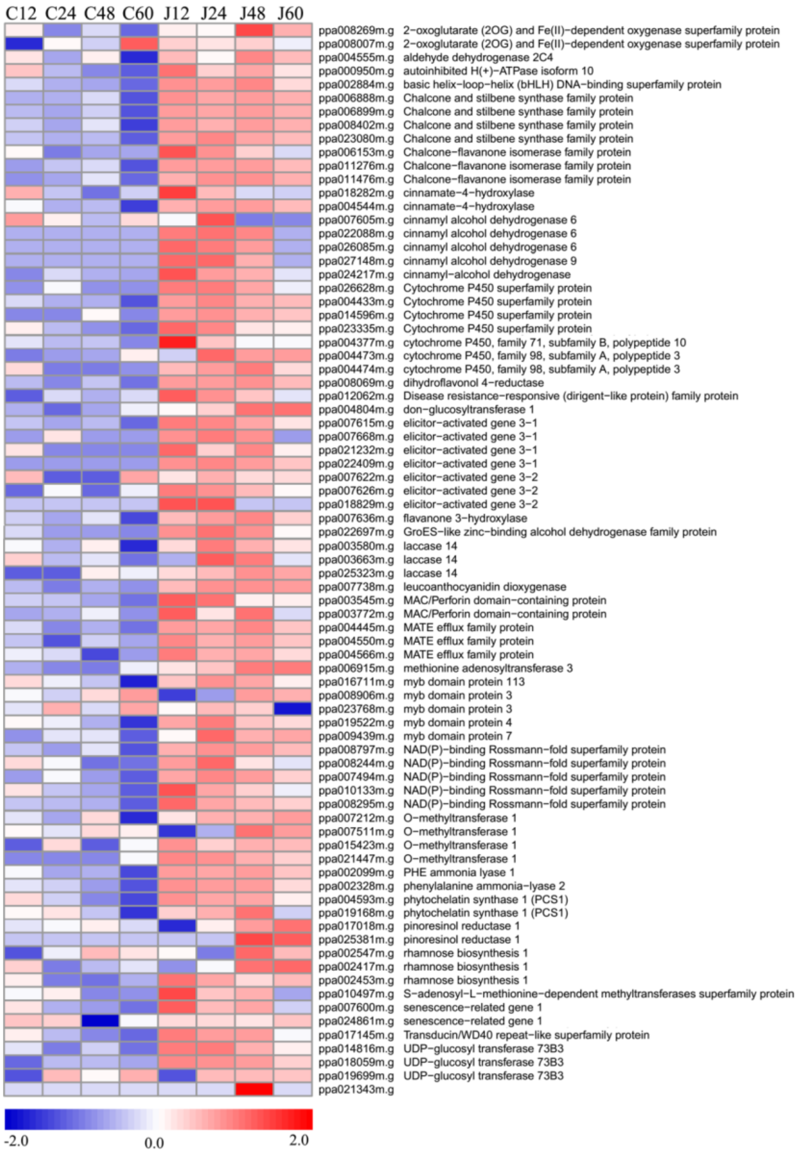

Supplementary Figure 2 Heat map diagram of expression levels of DEGs involved in phenylpropanoid biosynthesis and metabolism. Data for gene expression level were normalized to z-score by row.
