# Supplementary material for: Gene Expression Changes during the Gummosis Development of Peach Shoots in Response to Lasiodiplodia theobromae Infection Using RNA-Seq
Source: Front Physiol. 2016 May 9;7:170. doi: 10.3389/fphys.2016.00170 (PMC4861008; doi:10.3389/fphys.2016.00170)
Supplement: Supplementary file 13 [file Image3.PDF]

Supplementary Figure

Gene expression changes during the gummosis development of peach shoots in response to *Lasiodiplodia theobromae* infection using RNA-Seq

Lei Gao[1], Yuting Wang[2], Zhi Li[3], He Zhang[4], Junli Ye[5] and Guohuai Li[6]*
*Corresponding author: Guohuai Li; E-mail address: liguohuai@mail.hzau.edu.cn

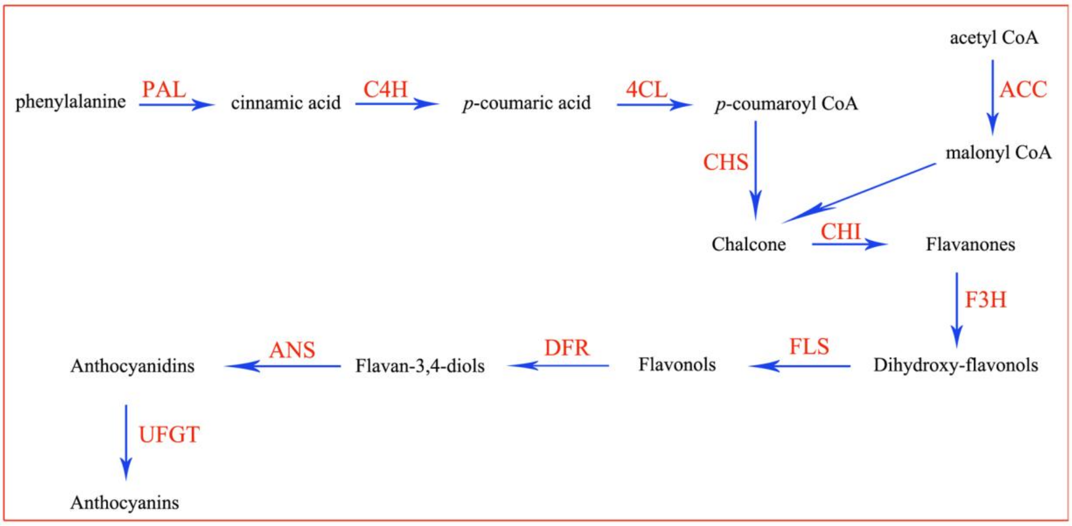

Supplementary Figure 3 Overview of genes involved in anthocyanin biosynthetic pathway. ACC, acetyl-CoA carboxylase; PAL, phenylalanine ammonia-lyase; C4H, cinnamate 4-hydroxylase; 4CL, 4-coumarate:CoA ligase; CHS, chalcone synthase; CHI, chalcone isomerase; F3H, flavanone 3-hydroxylase; FLS, flavonol synthase; DFR, dihydroflavonol-4-reductase; ANS, anthocyanidin synthase; UFGT, UDP-flavonoid glucosyltransferase.
