# Supplementary material for: Gene Expression Changes during the Gummosis Development of Peach Shoots in Response to Lasiodiplodia theobromae Infection Using RNA-Seq
Source: Front Physiol. 2016 May 9;7:170. doi: 10.3389/fphys.2016.00170 (PMC4861008; doi:10.3389/fphys.2016.00170)
Supplement: Supplementary file 14 [file Image4.PDF]

Supplementary Figure

Gene expression changes during the gummosis development of peach shoots in response to *Lasiodiplodia theobromae* infection using RNA-Seq

Lei Gao[1], Yuting Wang[2], Zhi Li[3], He Zhang[4], Junli Ye[5] and Guohuai Li[6]*
*Corresponding author: Guohuai Li; E-mail address: liguohuai@mail.hzau.edu.cn

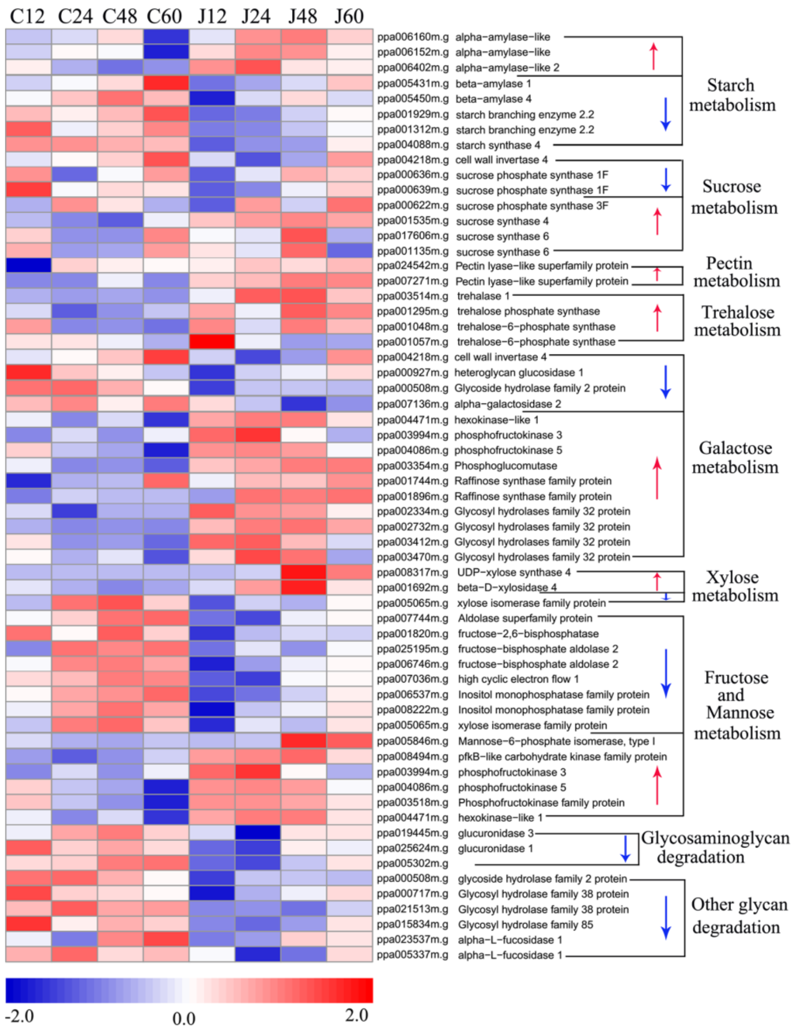

Supplementary Figure 4 Heat map diagram of expression levels of differentially expressed genes annotated in the carbohydrate metabolic pathways analyzed by KEGG. Data for gene expression level were normalized to z-score by row. Red and blue represent the upregulated and downregulated expression of these genes in the inoculated peach shoots, respectively.
