# Supplementary material for: Gene Expression Changes during the Gummosis Development of Peach Shoots in Response to Lasiodiplodia theobromae Infection Using RNA-Seq
Source: Front Physiol. 2016 May 9;7:170. doi: 10.3389/fphys.2016.00170 (PMC4861008; doi:10.3389/fphys.2016.00170)
Supplement: Supplementary file 16 [file Image6.PDF]

Supplementary Figure

Gene expression changes during the gummosis development of peach shoots in response to *Lasiodiplodia theobromae* infection using RNA-Seq

Lei Gao[1], Yuting Wang[2], Zhi Li[3], He Zhang[4], Junli Ye[5] and Guohuai Li[6]*
*Corresponding author: Guohuai Li; E-mail address: liguohuai@mail.hzau.edu.cn

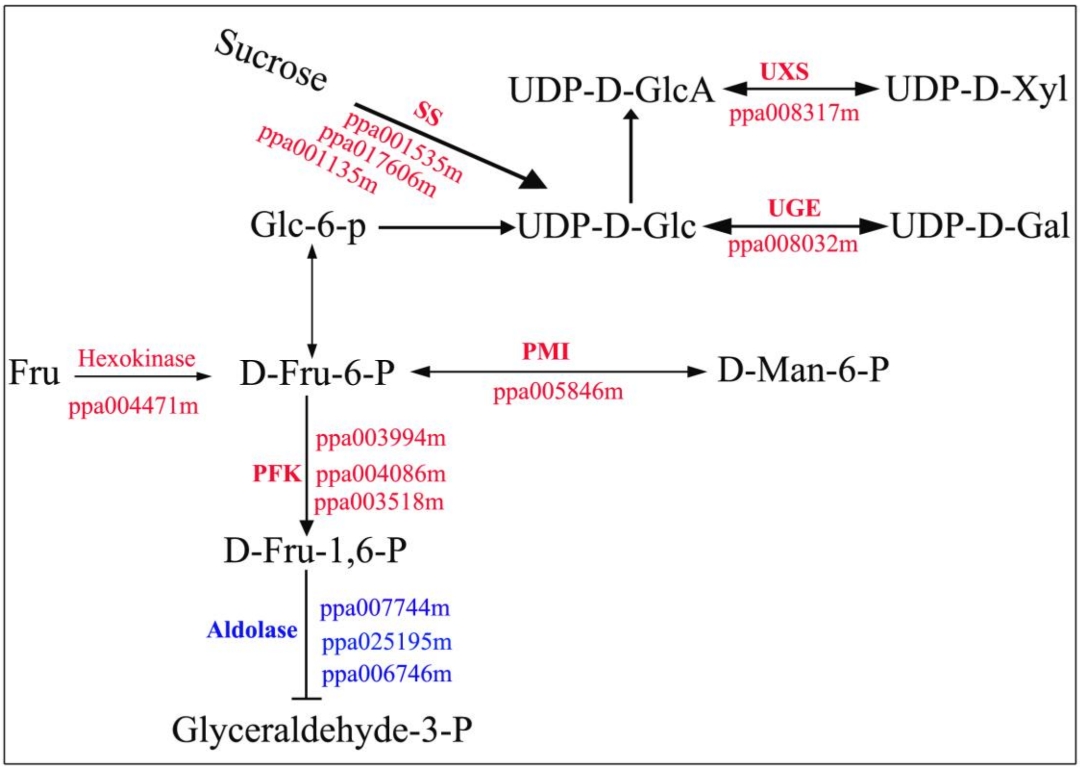

Supplementary Figure 6 Overview of genes involved in biosynthesis and metabolism of nucleotide sugar. SS, sucrose synthase; UGE, UDP-D-glucose 4-epimerase; UXS, UDP-xylose synthase; PMI, phosphomannose isomerase; PFK, phosphofructokinase; Glc, glucose; GlcA, glucuronic acid; Gal, galactose; Fru, fructose; Man, mannose; Xyl, xylose. Red and blue words represent the upregulated and downregulated genes, respectively. T-bar indicates inhibition.
