# Supplementary material for: Gene Expression Changes during the Gummosis Development of Peach Shoots in Response to Lasiodiplodia theobromae Infection Using RNA-Seq
Source: Front Physiol. 2016 May 9;7:170. doi: 10.3389/fphys.2016.00170 (PMC4861008; doi:10.3389/fphys.2016.00170)
Supplement: Supplementary file 17 [file Image7.PDF]

Supplementary Figure

Gene expression changes during the gummosis development of peach shoots in response to *Lasiodiplodia theobromae* infection using RNA-Seq

Lei Gao[1], Yuting Wang[2], Zhi Li[3], He Zhang[4], Junli Ye[5] and Guohuai Li[6]*
*Corresponding author: Guohuai Li; E-mail address: liguohuai@mail.hzau.edu.cn

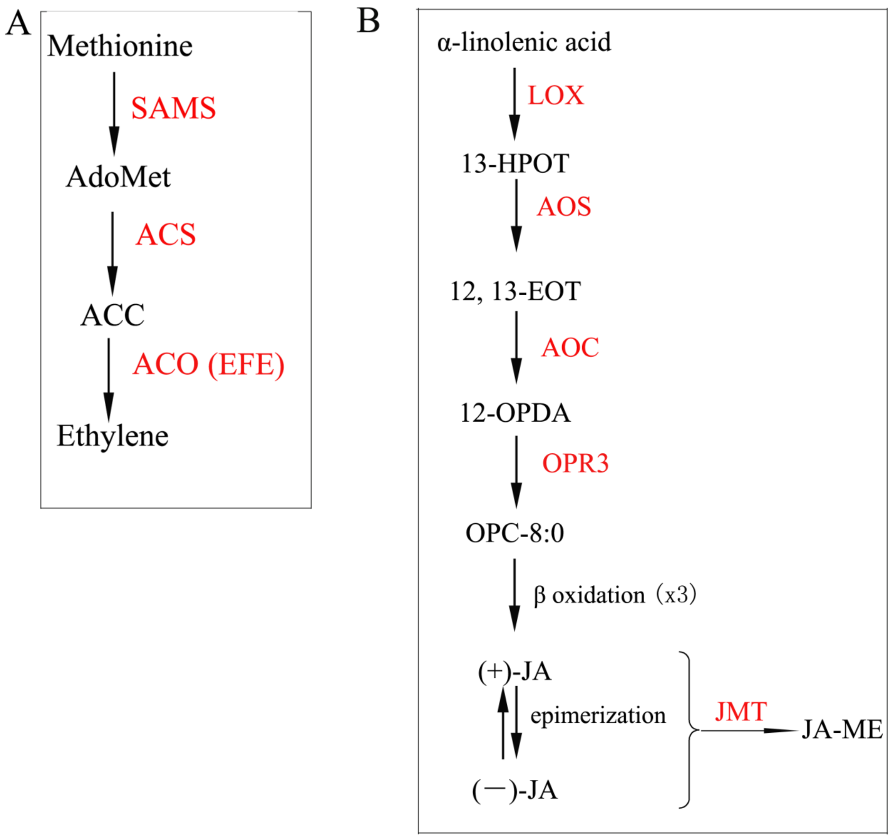

Supplementary Figure 7 Overview of genes involved in ethylene **(A)** and jasmonic acid **(B)** biosynthetic pathway, respectively. SAMS, S-adenosyl-methionine synthase; AdoMet, S-adenosyl-methionine; ACS, 1-aminocyclopropane-1-carboxylic acid (ACC) synthase; ACO, ACC oxidase; EFE, Ethylene-forming enzyme; LOX, lipoxygenases; 13-HPOT, 13(S)-hydroperoxyoctadecatrienoic acid; AOS, allene oxide synthase; 12, 13-EOT, 12, 13 (S)-epoxyoctade-catrienoic acid; AOC, allene oxide cyclase; 12-OPDA, 12-oxo-phytodienoic acid; OPR3, OPDA reductase3; OPC-8:0, 3-oxo-2-(2-(Z)-pentenyl)-cyclopentane-1-octanoic acid; JMT, jasmonic acid carboxyl methyltransferase
